# Elucidating the Anti-Diabetic Mechanisms of Mushroom Chaga (*Inonotus obliquus*) by Integrating LC-MS, Network Pharmacology, Molecular Docking, and Bioinformatics

**DOI:** 10.3390/ijms26115202

**Published:** 2025-05-28

**Authors:** Nidesha Randeni, Jinhai Luo, Yingzi Wu, Baojun Xu

**Affiliations:** Food Science and Technology Program, Department of Life Sciences, Beijing Normal-Hong Kong Baptist University, Zhuhai 519087, China; nidesha.randeni96@gmail.com (N.R.); luojinhai@uic.edu.cn (J.L.); wuyingzi@uic.edu.cn (Y.W.)

**Keywords:** type 2 diabetes, insulin resistance, hyperglycemia, Chaga, glycolysis, natural products

## Abstract

Diabetes mellitus is characterized by insulin resistance, impaired glucose homeostasis, and dysregulated glucose metabolism, leading to complications. *Inonotus obliquus* (Chaga) has shown potential anti-diabetic effects, but the bioactive compounds and molecular targets remain unclear. This study aimed to identify the bioactive components of Chaga and elucidate their anti-diabetic mechanisms using LC-MS compound screening, network pharmacology, molecular docking, and bioinformatics analyses. Chaga extract was prepared using 95% ethanol, and bioactive compounds were identified through UHPLC-QE-MS analysis. Target prediction was conducted using Swiss Target Prediction and SEA databases, while diabetes-related targets were retrieved from GeneCards. A PPI network was constructed using STRING and analyzed for GO and KEGG enrichment. Molecular docking was performed using AutoDock Vina, and gene expression was validated using the GSE7014 dataset and GEPIA database, with immune cell infiltration analyzed through CIBERSORT. UHPLC-QE-MS identified 30 bioactive compounds from Chaga, including 21 triterpenoids, four flavonoids, and two diterpenoids. Network pharmacology predicted 432 anti-diabetic targets, with 167 core targets enriched in key pathways, primarily the PI3K/Akt signaling pathway. Molecular docking revealed strong binding affinities of five key compounds with seven core targets. Bioinformatics analysis validated significant expression changes in ESR1, IL6, and SRC, while immune cell infiltration analysis showed correlations between core targets and immune cell subtypes. This study highlights the anti-diabetic potential of Chaga by identifying key bioactive compounds and their interactions with central diabetic targets. Further in vitro and in vivo studies are needed to validate these findings.

## 1. Introduction

Diabetes mellitus (DM) is a chronic metabolic condition marked by sustained hyperglycemia resulting from insulin secretion deficiency, insulin resistance, or a combination of both. Type 2 diabetes (T2D) constitutes more than 90% of all diabetes cases and is a primary contributor to morbidity and mortality globally. The International Diabetes Federation (IDF) estimates that by 2045, 783 million adults (20–79 years old) will be living with diabetes, up from around 537 million today [1]. DM raises the risk of consequences like cardiovascular illnesses, nephropathy, neuropathy, and retinopathy, which present a significant economic burden, with worldwide healthcare costs surpassing USD 966 billion in 2021 [1].

The pathophysiology of diabetes is largely influenced by glucose metabolism, which is modulated by insulin. Under normal conditions, insulin binds to its receptor. It activates complex signaling pathways, including phosphatidylinositol 3-kinase/protein kinase B (PI3K/Akt), resulting in the translocation of glucose transporter type 4 (GLUT4) to the cell membrane. This facilitates glucose uptake into muscle and adipose tissues. However, in T2D, insulin resistance impairs this signaling cascade, resulting in decreased glucose uptake and hyperglycemia [2]. In addition to glucose uptake, the PI3K/Akt pathway regulates glycogenesis (glycogen synthesis) and gluconeogenesis (glucose production). Protein kinase B (Akt) inhibits glycogen synthase kinase-3β (GSK-3β), resulting in glycogen synthase activation and promoting glycogen storage in the liver and muscle [3]. Furthermore, chronic hyperglycemia leads to the overproduction of reactive oxygen species (ROS) and advanced glycation end-products (AGEs), which contribute to β-cell dysfunction, insulin resistance, and inflammation. ROS promotes the non-enzymatic glycation of proteins and lipids, while AGEs trigger inflammatory responses, endothelial dysfunction, and vascular damage, contributing to complications such as diabetic nephropathy, neuropathy, and retinopathy [4]. The dysregulation of these pathways underscores the complexity of diabetes and highlights the need for multi-target therapeutic approaches, such as those offered by bioactive compounds from natural sources.

Chaga, or *Inonotus obliquus*, is a medicinal mushroom that grows mainly on birch trees in cold climates like China, Korea, Russia, and Northern Europe. It is widely used for its anti-cancer [5], antioxidant [6], anti-inflammatory [7], anti-viral, anti-fatigue [8], anti-diabetic [9], hepatoprotective, and immunomodulatory qualities [10]. Chaga possesses many bioactive compounds, such as triterpenoids, phenolics, and sterols, which support its broad variety of pharmacological effects [11]. Triterpenoids and polysaccharides are major phytochemicals in *I. obliquus* that play a significant role in its anti-diabetic properties. They boost insulin signaling through the PI3K/Akt pathway, increase GLUT4 translocation, activate AMP-activated protein kinase (AMPK) to enhance glucose absorption, and decrease inflammation and oxidative stress, among other ways [12,13,14].

Despite the promising therapeutic potential of Chaga, the specific bioactive compounds responsible for its anti-diabetic effects remain largely unexplored. Modern analytical techniques such as liquid chromatography–mass spectrometry (LC-MS) provide a powerful tool for identifying and characterizing bioactive compounds in Chaga. Moreover, two system-based approaches, network pharmacology and molecular docking, enable the prediction of molecular targets and pathways associated with these compounds. It facilitates a comprehensive understanding of their multi-target mechanisms. A combined approach of LC-MS, network pharmacology, molecular docking, and bioinformatics allows for a systematic investigation of Chaga’s phytochemicals in diabetes management.

This study aims to determine the anti-diabetic mechanisms of Chaga by integrating LC-MS for compound identification, network pharmacology for target prediction and pathway analysis, molecular docking to explore their binding affinity, and bioinformatics to validate the results. The graphical abstract provides an overview of the study design and methodology, illustrating the integrative workflow from compound identification to target-pathway analysis. Specifically, we seek to characterize the phytochemicals present in Chaga, analyze their interactions with key signaling pathways, and evaluate their potential to regulate glucose metabolism. By uncovering the molecular interactions and pharmacological mechanisms, this research will provide valuable insights into the therapeutic applications of Chaga-derived phytochemicals for diabetes management, paving the way for future clinical applications.

## 2. Results

### 2.1. Phytochemicals Derived from Chaga

The constituents of Chaga were examined using the UHPLC-QE-MS technique. Based on the sample’s mass spectrum and the commercial MS database (BIOTREE TCM database), which included the acquisition mode, retention time, MS spectra, adduct ions of [M+H]^+^, [M-H]^−^, M+HCOO, M+CH_3_COO, and [M+FA-H]^−^, isotope information, and secondary fragment information, the identification was completed. A total of twenty-one triterpenoid compounds, two diterpenoids, and four flavonoids were identified (Table 1). These triterpenoid compounds include the following: alisol A, asiatic acid, arjungenin, dehydrotumulosic acid, euscaphic acid, colosolic acid, laetiposide G, 2-[6-(2-carboxyethyl)-7-ethenyl-3a,6,9b-trimethyl-1,2,3,4,7,8-hexahydro cyclopenta[a]naphthalen-3-yl]-6-methyl-5-methylideneheptanoic acid, betulinic acid, oleanonic acid, mimusopsic acid, neokadsuranic acid B, smilagenone, lupenone, 18 beta-glycyrrhetintic acid, 3beta-hydroxy-21-oxo-11,13(18)-oleanadien-28-oic acid methyl ester, wilforlide A, dehydrotrametenolic acid, betulin, 2-methyl-6-(4,4,10,13,14-pentamethyl-3,11-dioxo-2,5,6,7,12,15,16,17-octahydro-1H-cyclopenta[a]phenanthren-17-yl)hept-2-enal, and methyl 4-(12-hydroxy-4,4,10,13,14-pentamethyl-3,7,11,15-tetraoxo-2,5,6,12,16,17-hexahydro-1H-cyclopenta[a]phenanthre-17-yl)pentanoate. Isosteviol and forskolin are the two diterpenoids. The compounds belonging to the flavonoid group, tamarixetin (isoflavone), tectorigenin (flavone), epicatechin (flavanol), and glabrol (flavanone) were identified. Apart from them, one triterpenoid glucoside (terminolic acid) and one triterpenoid sapogenin (panaxatriol) were also identified. 2-[6-(2-carboxyethyl)-7-Ethenyl-2-hydroxy-3a,6,9b-trimethyl-1,2,3,4,7, 8-hexahydro cyclopenta[a]naphthalen-3-yl]-6-hydroxy-6-methyl-5-methylideneheptanoic acid can be considered Polycyclic Aromatic Hydrocarbons (PAHs).

### 2.2. Diabetes and Compound-Related Targets

The Swiss Target Prediction [15] and SEA databases [16] were used to search for targets based on the structures of the 30 bioactive chemicals found in Chaga. After removing duplicates, 541 targets were discovered. Targets associated with the disease were gathered from the GeneCards database [17]. A total of 8321 disease-related targets were retrieved for additional study after duplicates were eliminated. Additionally, 5614 targets related to glucose metabolism were gathered from the GeneCards database.

### 2.3. Anti-Diabetes Targets of Chaga and PPI Analysis

**Figure 1:**
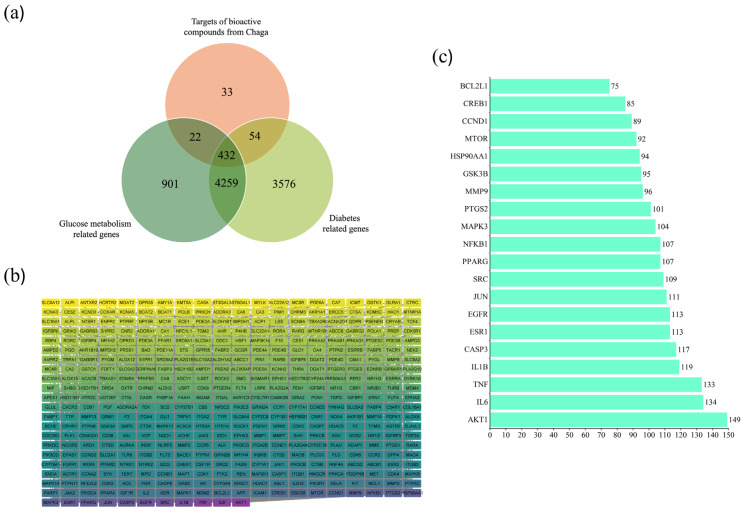
(**a**) The Venny figure between the targets of Chaga and diabetic-related targets that enhance glucose metabolism. (**b**) PPI network of 431 overlapping genes drawn by String database. (**c**) The top 20 anti-diabetic targets of Chaga ranked by the degree values of the PPI network.

Including the 432 interacting targets, Figure 1a displays the Venn diagram between the drug target, diabetes, and glucose metabolism [18]. These are the possible anti-diabetic targets of Chaga, which enhance the metabolism of glucose.

To analyze protein–protein interactions, these genes were loaded into the String database (Figure 1b) [19]. With 431 nodes and 8021 edges, the PPI network illustrates the network’s complexity. The average node degree and local clustering coefficient for every node in the PPI network were 37.22 and 0.47, respectively. The PPI network had an enrichment *p*-value of less than 1.0 × 10^−16^. The findings demonstrated a significant association between the PPI network’s nodes. Figure 1c shows the proteins in this network with the highest 20-degree value.

### 2.4. Core Targets for Enrichment Analysis

**Figure 2:**
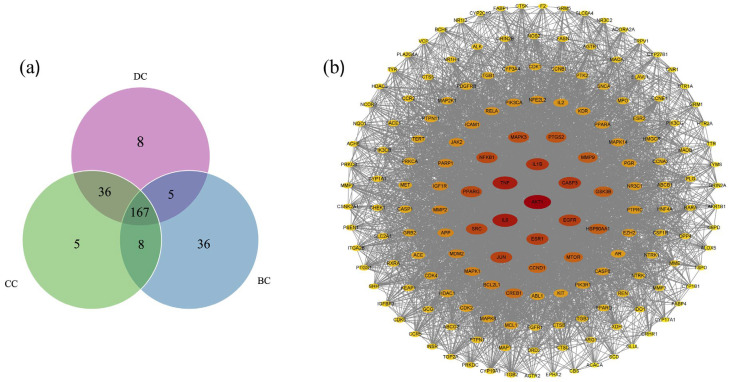
(**a**) The Venny figure between the BC, CC, and DC. (**b**) The PPI network of the 167 core targets.

According to the PPI network, the medians of DC, CC, and BC were 27, 0.4638, and 146.54, respectively. A total of 167 core targets were identified, each meeting or exceeding the median values for betweenness, closeness, and degree. The results are illustrated in the Venn diagram (Figure 2a). After importing core targets into the Cytoscape software (version 3.10.1), an interaction network was constructed (Figure 2b). The nodes’ edges were shown by the DC, where a higher degree indicated a greater association with other nodes. The CC of a node measured its distance from others, with nodes positioned more centrally in the network when their CC value approached 1. BC demonstrated a node’s significance and convenience. More association with others was indicated by a higher degree of betweenness. Nodes in the internal cycle had a higher degree of centrality than those in the exterior cycle. Each node’s color changed from red to yellow as its degree decreased.

### 2.5. Construction of Central Targets of Chaga for Component–Target Docking

**Figure 3:**
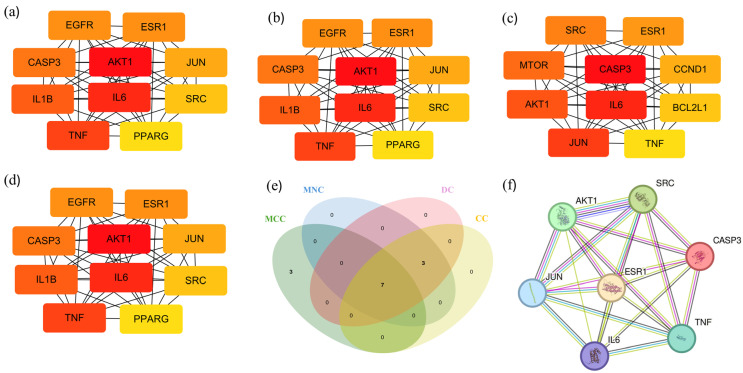
(**a**) The network of targets with the top 10 CC. (**b**) The network of targets with the top 10 DCs. (**c**) The network of targets with the top 10 MCCs. (**d**) The network of targets with the top 10 MNCs. (**e**) The Venny figure between degree, closeness, MCC, and MNC. (**f**) The network of central targets.

The network of targets, each ranking in the top 10 for DC, CC, MCC, and MNC, was selected for molecular docking. In order to obtain the overlapping targets of four networks as central targets, including AKT1, ESR1, CASP3, EGFR, JUN, SRC, and TNF, the Venny online tool was used (Figure 3e). STRING 12.0 was used to visualize these central nodes [11] (Figure 3f). The network comprised 21 edges, with an average node degree of six and an average local clustering coefficient of one, suggesting full interconnectivity among the nodes.

### 2.6. GO Enrichment Analysis of Core Targets

**Figure 4:**
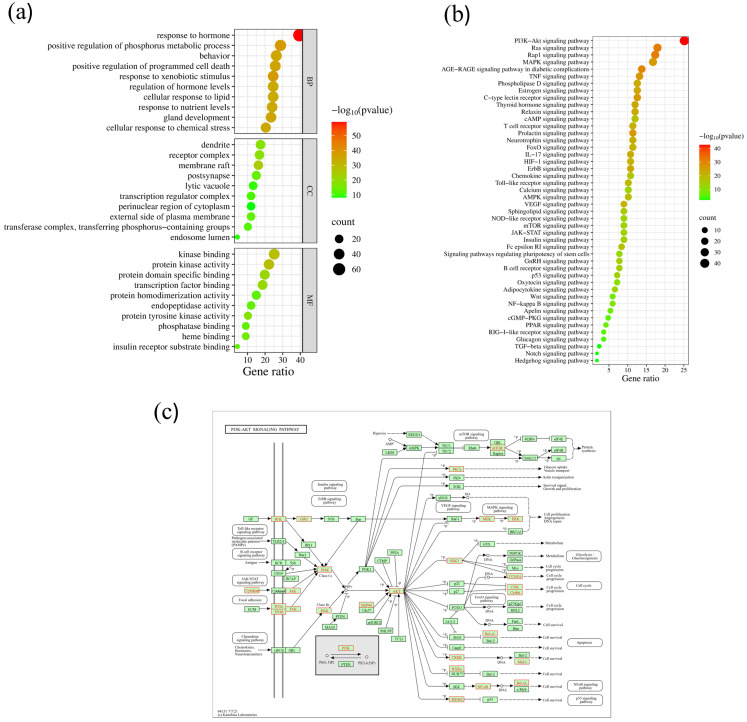
(**a**) The GO analysis of 167 anti-diabetic core targets, including the top 10 items of BPs, CCs, and MFs. (**b**) The KEGG pathway analysis of 167 anti-diabetic core targets. (**c**) PI3K/Akt signaling pathway.

The GO analysis of BPs, CCs, and MFs was carried out by Metascape [20]. A total of 167 core targets were associated with 1983 BP terms, 131 CC terms, 204 MF terms, and 206 KEGG pathway terms. For pathways and all enrichment analysis terms, the *p*-value was less than 0.01. At least three genes were enriched in each enrichment analysis term, and the *p*-value for each term was less than 0.01. A GO-enriched dot bubble, including the top 10 BPs, CCs, and MFs, is displayed in Figure 4a. In this enrichment analysis, the gene target involves multiple BPs, including response to hormones, positive regulation of the phosphorus metabolic process, behavior, positive regulation of programmed cell death, response to xenobiotic stimulus, regulation of hormone levels, cellular response to lipids, response to nutrient levels, etc. The top CCs included dendrite, receptor complex, membrane raft, postsynapse, lytic vacuole, transcription regulator complex, perinuclear region of cytoplasm, etc. Furthermore, the MFs included kinase binding, protein kinase activity, protein domain-specific binding, transcription factor binding, protein homodimerization activity, endopeptidase activity, etc.

### 2.7. KEGG Enrichment Analysis of Core Targets

The Metaspace database was used to perform the KEGG enrichment analysis [20]. Figure 4b displays the top 30 signaling pathways that were enriched out of 167 KEGG pathway keywords that met *p* < 0.05. The results of the KEGG pathway enrichment analysis indicated that the molecular mechanism of Chaga in treating diabetes may involve the PI3K/Akt signaling pathway, Ras signaling pathway, RAP1 signaling pathway, MAPK signaling pathway, etc. The main signaling pathway directly involved in glucose metabolism-induced diabetes is the PI3K/Akt signaling pathway (Figure 4c). Chaga’s beneficial effects on diabetes may be mediated through this signaling system.

### 2.8. Construction of Chaga-Components-Targets-Diabetes-Signaling Pathway Network

**Figure 5:**
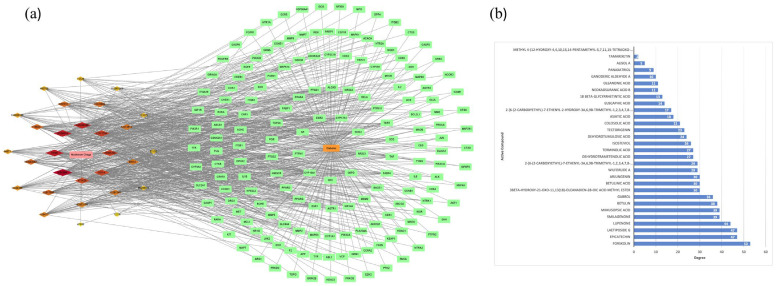
(**a**) The network of drug-compound–target-disease. The pink node represents the Chaga. The red-yellow nodes represent the phytochemicals derived from Chaga. The green nodes represent the anti-diabetic targets of bioactive phytochemicals. The orange node represents diabetes. (**b**) The degree value of the bioactive phytochemicals derived from Chaga.

Using 167 main targets and 30 plant components from Chaga, a network of phytochemicals-diabetes was built (Figure 5a). The network had 197 nodes and 915 edges, according to the results. The relationship between anti-diabetic core targets and active phytochemicals is represented by each edge. Each phytochemical’s degree value determines the size of its nodes, which grow in size from small to large as the nodes’ degrees rise. The degree of a node in a network is a representation of its core level and the number of edges connecting it to other nodes [21]. The degree values of all 30 phytochemicals from Chaga in this network are presented in Figure 5b.

### 2.9. Molecular Docking

**Figure 6:**
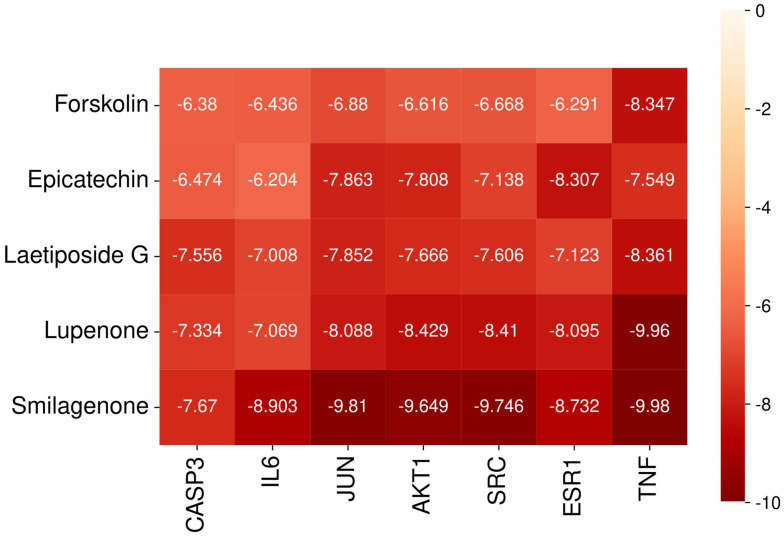
The heat map of the molecular docking result between the central targets and the active phytochemicals from Chaga.

Molecular docking analysis was performed on five phytochemicals from Chaga with the highest degree values, targeting seven key anti-diabetic proteins. All selected compounds exhibited negative binding energies, suggesting their potential for interaction with the target proteins. To facilitate visual interpretation, a hit map was generated (Figure 6). The binding affinity (kcal/mol) reflects the strength of molecular interactions, where lower values indicate stronger binding. According to Wong et al. [22], a binding affinity below −7.0 kcal/mol signifies strong binding, while values below −5.0 kcal/mol indicate moderate binding. Out of the 35 docking results, eight exhibited binding affinities below −5.0 kcal/mol. While the rest were less than −7 kcal/mol. The docking results demonstrated that all selected phytochemicals in Chaga effectively bind to key target proteins, including CASP3 (PDB ID: 1RE1) [23], IL6 (PDB ID: 1ALU) [24], JUN (PDB ID: 5T01) [23], AKT1 (PDB ID: 4EJN) [25], SRC (PDB ID: 1Y57) [26], ESR1 (PDB ID: 7UJO) [24], and TNF (PDB ID: 2AZ5) [27] indicating a strong interaction potential.

**Figure 7:**
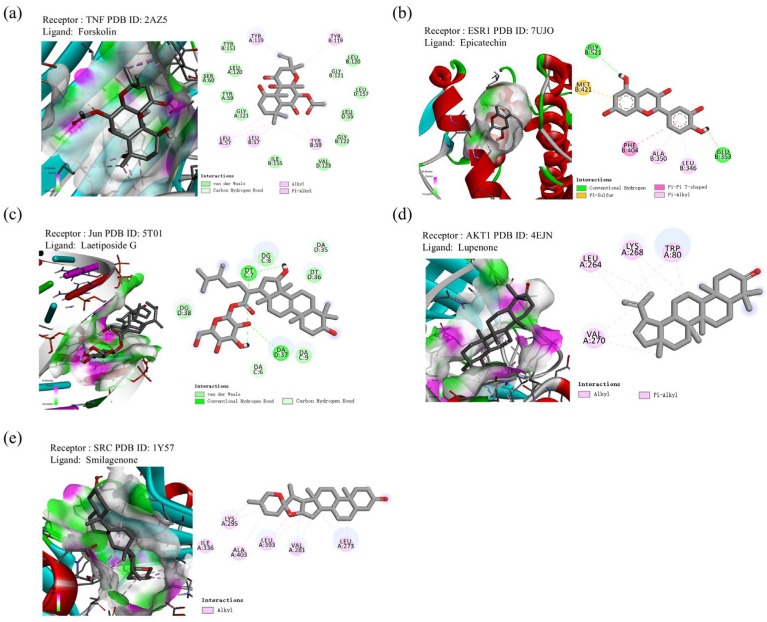
The 2D and 3D diagrams of the docking result between the central targets and the active phytochemicals from Chaga (**a**) TNF-Foskolin complex. (**b**) ESR1-Epicatechin complex (**c**), JUN-Laetiposide G complex (**d**), AKT1-Lupenone complex (**e**), SRC-Smilagenone complex.

Part of the 3D and 2D results of some docking complexes with strong binding affinity are shown in Figure 7, including the TNF-Foskolin complex (−9.98 kcal/mol), ESR1-Epicatechin complex (−8.307 kcal/mol), JUN-Laetiposide G complex (−7.852 kcal/mol), AKT1-Lupenone complex (−8.429 kcal/mol), and SRC-Smilagenone complex (−9.746 kcal/mol). The TNF-Foskolin complex had five hydrophobic interactions (alkyl and Pi-Alkyl interactions) with TYR(A:119), TYR(B:119), TYR(B:59), LEU(A:57), and LEU(B:57), one carbon–hydrogen bond with GLY(B:121), and some van der Waals forces. The ESR1-Epicatechin complex was stabilized by conventional hydrogen bonds with GLY(B:521) and GLU(B:353), one Pi-Pi T-shaped interaction with PHE(B:404), and two Pi-Alkyl interactions with ALA(B:350) and LEU(B:346). MET(B:421) interacts with the aromatic ring of epicatechin via a Pi-Sulfur interaction. JUN-Laetiposide G complex presented two carbon–hydrogen bonds with DA(D:35) and DA(C:6), two conventional hydrogen bonds with DA(D:37) and DT(C:7), and some van der Waals forces. The stability of the AKT1-Lupenone complex was based on alkyl and Pi-Alkyl interactions with LEU(A:264), LYS(A:268), TRP(A:80), and VAL(A:270). SRC-Smilagenone complex interacts with LYS(A:295), ILE(A:336), ALA(A:403), LEU(A:393), VAL(A:281), and LEU(A:273) through alkyl interactions.

### 2.10. Bioinformatics

**Figure 8:**
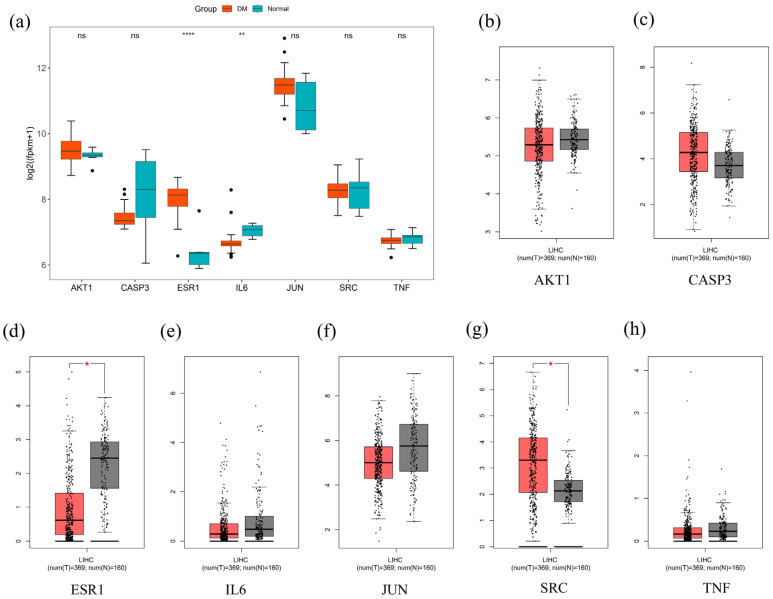
Core gene expression analysis. (**a**) Core genes (AKT1, CASP3, ESR1, IL6, JUN, SRC, and TNF) expression in the training dataset (GSE7014), green color indicates healthy people and red color represents diabetes people. (**b**–**h**) Core genes (AKT1, CASP3, ESR1, IL6, JUN, SRC, and TNF) expression in liver hepatocellular carcinoma dataset of GEPIA database, red color indicates liver cancer people while gray color represents healthy people. “*” indicates *p*-value < 0.05, “**” indicates *p*-value < 0.01, “****” indicates *p*-value < 0.0001.

In the GSE7014 dataset, the expression of ESR1 was significantly increased, and the expression of IL6 was significantly reduced in diabetes patients (Figure 8a). When exploring the core genes’ expression in the GEPIA database (Figure 8b–h), we found that ESR1 gene expression was significantly reduced in liver cancer patients, while SRC gene expression was significantly increased in liver cancer compared to healthy people.

**Figure 9:**
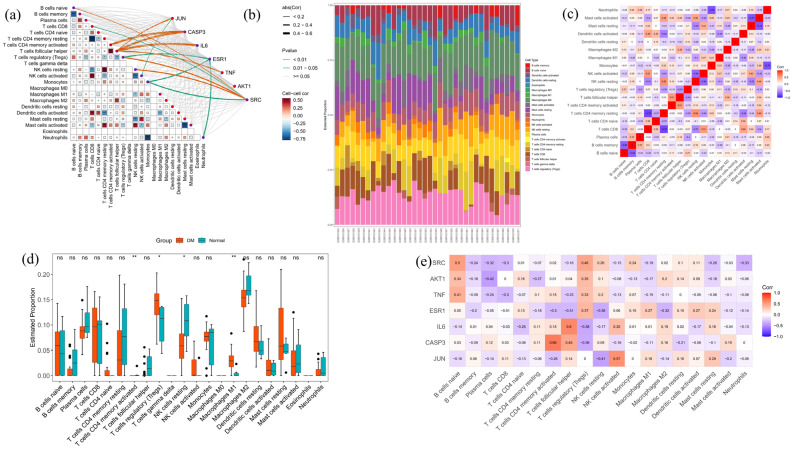
Immune cell infiltration analysis. (**a**) Core genes and 22 types of immune cell correlation heatmap. The correlation was calculated using the Pearson method, and the correlation between core genes and different immune cells was represented by connecting lines. The orange line represents *p*-values < 0.01, and the thickness of the line represents the absolute value of the correlation. (**b**) The relative abundance of 22 types of immune cells, with the X-axis representing different sample names. (**c**) The heatmap shows the correlation between 22 types of immune cells, calculated using the Pearson method, with darker colors indicating a higher correlation. (**d**) Differential expression of 22 types of immune cells in diabetes and normal groups. (**e**) The heatmap showing the correlation between the expression levels of 22 types of immune cells and core genes was calculated using the Pearson method. The darker the color, the higher the correlation. “*” indicates *p*-value < 0.05, “**” indicates *p*-value < 0.01, “***” indicates *p*-value < 0.001.

CIBERSORT is a proper algorithm for exploring the correlations between characteristic genes and immune cells. Using CIBERSORT, the mechanisms linking the feature genes to immune cell infiltration in diabetes were explored. The network diagram of the correlation between different types of immune cells and specific genes is shown in Figure 9a. The absolute values of the correlation between the CASP3 gene and T cell CD4 memory activated and T cell follicular helper are between 0.4 and 0.6. The absolute value of the correlation between the IL6 gene and T cell follicular helper is between 0.4 and 0.6, making it the gene with the highest correlation in the Mantel test. The relative abundance of 22 types of immune cells is shown in Figure 9b. The content of T cell regulatory (Tregs) cells in DM was higher than that in the normal group, with statistical differences. Figure 9c is the correlation heatmap between immune cells, and Figure 9e is the correlation heatmap between immune cells and gene expression levels, with numerical values representing correlation coefficients. The correlation between the two images was calculated using the Pearson method. The correlation between SRC and naïve B cells is the highest, with a correlation coefficient of 0.5. ATK1 has the highest correlation with plasma cells, with a correlation coefficient of −0.42. The correlation between TNF and B-cell naïve is the highest, with a correlation coefficient of 0.41. ESR1 has the highest correlation with T cell regulators (Tregs), with a correlation coefficient of 0.37. The correlation between IL6 and T cell follicular helper is the highest, with a correlation coefficient of 0.6. CASP3 has the highest correlation with T cell CD4 memory activated, with a correlation coefficient of 0.66. JUN and NK cell activation have the highest correlation, with a correlation coefficient of 0.57.

## 3. Discussion

*Inonotus obliquus* (Chaga) is an herbal medicinal fungus with a wide range of therapeutic potential [28]. The present study provides a comprehensive study into the possible anti-diabetic potential of *I*. *obliquus* (Chaga) through the combination of LC-MS, network pharmacology, molecular docking, and bioinformatics. The bioactive ingredients in fruits, cereals, nuts, and herbal medicines were frequently identified using LC-MS [8,29,30,31]. Network pharmacology and molecular docking are often integrated to uncover the molecular mechanisms underlying disease treatment and to elucidate compound–target interactions [32,33]. The LC-MS analysis identified various bioactive compounds, including triterpenoids, diterpenoids, flavonoids, and triterpenoid derivatives; among them, triterpenoids were the majority. In total, they possessed 432 anti-diabetic targets. A total of 5 out of the 30 bioactive compounds were chosen for molecular docking because their anti-diabetic targets surpassed the average mean value.

Among the 432 anti-diabetic targets, 431 were identified by the String database, allowing for the construction of a PPI network. A total of 167 targets had BC, CC, and DC values above the median and were identified as core targets for GO and KEGG enrichment analysis. Among these, seven key targets (JUN, AKT1, ESR1, CASP33, TNF, SRC, and IL6) were found to have the highest network connectivity, indicating their central role in treating diabetes with Chaga. These targets were identified as key targets for molecular docking analysis, indicating their central role in diabetes-related pathways.

The GO enrichment analysis revealed that the identified gene targets are involved in multiple BPs, suggesting their broad functional relevance in glucose metabolism and diabetes regulation. Key processes such as hormone response, regulation of hormone levels [34], phosphorus metabolic regulation [35], and programmed cell death [36] indicate the potential role of *Inonotus obliquus*-derived bioactive compounds in modulating insulin signaling, glucose metabolism, and energy homeostasis. The CCs’ analysis highlighted the localization of these targets in structures such as dendrites, receptor complexes, membrane rafts, and transcription regulator complexes, further emphasizing their role in signal transduction and metabolic regulation at various cellular sites. In terms of MFs, significant enrichment in kinase binding, protein kinase activity, transcription factor binding, and protein domain-specific binding highlights the involvement of these targets in key insulin signaling pathways. Given that kinases and transcription factors play critical roles in glucose uptake, insulin sensitivity, and inflammatory responses [37,38]. These findings suggest that bioactive compounds from *Inonotus obliquus* may exert anti-diabetic effects by modulating these molecular functions.

The KEGG pathway enrichment analysis revealed that the anti-diabetic effects of Chaga may be mediated through multiple signaling pathways, including PI3K/Akt, Ras, RAP1, and MAPK signaling pathways. Among these, the PI3K/Akt signaling pathway plays a direct and crucial role in regulating glucose metabolism and is a key target for diabetes treatment. This pathway is essential for insulin signaling and facilitating glucose uptake, glycogen synthesis, and lipid metabolism. Dysregulation of PI3K/Akt signaling contributes to insulin resistance and impaired glucose homeostasis, which are hallmarks of T2D [39,40]. The PI3K/Akt pathway is primarily activated by insulin binding to its receptor, leading to a cascade of phosphorylation events that enhance GLUT4 translocation to the cell membrane, allowing glucose uptake into cells. When this pathway is disrupted, glucose uptake is significantly impaired, leading to hyperglycemia [39,41]. Additionally, PI3K/Akt signaling influences glycogenesis [42] and gluconeogenesis [43] by modulating key enzymes, helping maintain normal blood glucose levels. Overall, the GO and KEGG enrichment analysis provides strong evidence that the bioactive components of Chaga target multiple pathways and molecular mechanisms associated with diabetes, reinforcing its potential as a natural therapeutic agent. Future experimental validation through in vitro and in vivo studies is essential to further substantiate these computational findings and establish specific molecular mechanisms.

The degree value analysis indicated that certain compounds exhibited a higher interaction with core targets, suggesting their pivotal role in modulating glucose metabolism and insulin signaling. Among them, forskolin, epicatechin, lupenone, laetiposide G, and smilagenone had the highest degree values, implying their strong bioactivity in the anti-diabetic mechanism of Chaga. Forskolin has been previously linked to glucose homeostasis and insulin sensitivity [44,45], while epicatechin is well documented for its role in reducing oxidative stress and enhancing insulin function [46,47]. Lupenone, a triterpenoid, has shown potential effects on insulin resistance and glucose metabolism, though studies in this area remain limited [48]. Similarly, smilagenone and laetiposide G have been identified as bioactive compounds, but their precise role in diabetes management remains largely unexplored, highlighting the need for further experimental validation and mechanistic studies.

The Chaga-derived phytochemicals with the highest degree values (forskolin, epicatechin, lupenone, laetiposide G, and smilagenone) demonstrated strong interactions with the seven key targets (CASP3, IL6, JUN, AKT1, SRC, ESR1, and TNF), most compounds exhibiting binding affinities below −7 kcal/mol, indicating high binding stability. The molecular docking analysis revealed significant interactions between selected bioactive compounds and key protein targets involved in metabolic pathways. C-Jun can influence the expression of genes involved in the metabolic regulation of insulin resistance [49]. The JUN-Laetiposide G complex exhibited strong hydrogen bonding and van der Waals interactions, suggesting its potential to modulate JUN-related signaling in diabetes. The AKT1-Lupenone complex demonstrated pi-alkyl and alkyl interactions, indicating its possible role in regulating the PI3K/AKT pathway, a crucial target in glucose metabolism [50]. The SRC-Smilagenone complex showed strong hydrophobic interactions with SRC, implying its potential in glucose metabolism.

The GSE7014 dataset analysis revealed a significant increase in ESR1 expression and a decrease in IL6 expression in diabetes patients, suggesting their potential roles in metabolic dysregulation and inflammation. However, the GEPIA database showed downregulation of ESR1 in liver cancer, indicating tissue-specific differences in its regulation. This reflects the diverse role of ESR1 on tissue context, which acts as a metabolic regulator in diabetes, potentially improving insulin sensitivity and glucose homeostasis through estrogen signaling effects on pancreatic beta-cells and adipose tissue [51] but can function as a tumor promoter in cancers like hepatocellular carcinoma [52]. These findings highlight the complex involvement of ESR1 and IL6 in diabetes, warranting further investigation into their functional implications in metabolic disorders. Immune infiltration analysis using CIBERSORT demonstrated significant correlations between key diabetic genes and immune cells. Notably, CASP3 correlated strongly with activated CD4 memory T cells (r = 0.66) and IL6 with T follicular helper cells (r = 0.6), suggesting their role in immune modulation. SRC showed the highest correlation with naïve B cells (r = 0.5), while AKT1 was negatively correlated with plasma cells (r = −0.42), indicating potential immune dysregulation. These findings suggest that Chaga-derived compounds may exert anti-diabetic effects by targeting key genes involved in immune regulation and inflammation, providing further justification for experimental validation.

This study combines network pharmacology, molecular docking, and bioinformatic analysis to establish a theoretical basis for Chaga’s anti-diabetic mechanisms. However, the lack of experimental validation and data on bioavailability, pharmacokinetics, and toxicity remains a key limitation. Therefore, in vitro and in vivo investigations are necessary to validate these findings and confirm the biological significance of the identified phenolics. These studies will be crucial in assessing their real physiological effects and potential as therapeutic agents for obesity management.

## 4. Methodology

### 4.1. Preparation of Chaga Extracts

The Chaga sample was purchased from an e-commerce platform (Tmall Global) in China. It was collected in Siberia, Russia, then stored in Beijing Normal Hong Kong Baptist University, Food Science Laboratories, T8-508, where a voucher specimen (ID: UIC-FS-2024-06-01) was kept. Extraction of Chaga was carried out according to the methods described by Luo et al. [29], Liu et al. [24], and Zhang et al. [53], with some modifications. A total of 250.0 g of dried and powdered Chaga was extracted twice with 95% ethanol in a 1:10 solid–liquid ratio for 8 h. The resultant mixture was combined and filtered. The mixture was combined and filtered, and the filtrate was then concentrated using a rotary evaporator (Shanghai Yarong Biochemistry Instrument Factory, RE-52AA, Shanghai, China) to remove the ethanol content. The obtained aqueous solution was passed through an AB-8 resin column (Shanghai Yuanye Bio-Technology Co., Ltd., Shanghai, China). The elution of Chaga extract was performed using ethanol and was collected until no visible color was observed, indicating complete elution. The eluent was freeze-dried at −80 °C (Shanghai Yarong Biochemistry Instrument Factory, RE-52AA, Shanghai, China).

### 4.2. Ultra-High-Performance Liquid Chromatography–Q-Exactive HF Mass Spectrometry (UHPLC-QE-MS) Analysis

The application of UHPLC-QE-MS facilitated the analysis and identification of the Chaga compounds. After combining the Chaga with an 80% methanol (CNW Technologies, Dusseldorf, Germany) solution in water, it was centrifuged for 15 min at 4 °C at a velocity of 10,614× *g*. A microporous membrane with a pore size of 0.22 μm was used to filter the supernatant. LC-MS/MS analysis was conducted using an ultra-high-performance liquid chromatography (UHPLC) system (Vanquish, Thermo Fisher Scientific, Waltham, MA, USA.) equipped with a Waters UPLC BEH C18 column (1.7 μm (particle size), 2.1 mm × 100 mm). A 5 μL sample was injected with a flow rate of 0.5 mL/min. The mobile phase comprised 0.1% formic acid in water (solvent A) and 0.1% formic acid in acetonitrile (solvent B) (CNW Technologies, Dusseldorf, Germany). Gradient elution followed a multi-step linear program: starting at 85% A, decreasing to 25% A over 11 min, then to 2% A by the 12th minute, maintaining 2% A until the 14th minute, rapidly increasing to 85% A within 0.1 min, and holding at 85% A for the final 2 min.

In the mass spectrometry (Thermo Fisher Scientific, Waltham, MA, USA.), MS and MS/MS data were collected using a Q Exactive Focus mass spectrometer linked to Xcalibur- Massachusetts, USA 4.1 software in ion-driven acquisition (IDA) mode. The top three precursor ions from each cycle were chosen for additional fragmentation and analysis when the mass range scanned was between 100 and 1500 m/z. The capillary temperature was kept at 400 degrees Celsius, the sheath gas flow rate was set at 45 arbitrary units (Arb), and the auxiliary gas flow rate was set at 15 Arb. The resolution was set to 70,000 for full MS and 17,500 for MS/MS. In neutral collision energy (NCE) mode, the collision energy was adjusted to three different levels: 15, 30, and 45. In positive mode, the spray voltage was set to 4.0 kV, while in negative mode, it was set to −3.6 kV. The constituents of Chaga were identified and structurally elucidated using mass spectrometry, with their primary and secondary spectral data provided by the BIOTREE TCM database of Shanghai BIOTREE Biological Technology Co., Ltd. (Shanghai, China).

### 4.3. Protein Targets of Components Prediction

Network pharmacology studies were carried out according to the previously described method [24,29]. The PubChem database Smiles format of compounds from UHPLC-QE-MS analyses was entered into the Swiss Target Prediction (http://www.swisstargetprediction.ch/, accessed on 4 December 2024) and SEA (https://sea.bkslab.org/, accessed on 4 December 2024). With these two databases, compound targets based on a probability higher than zero were predicted [15,16]. The species for target prediction was *Homo sapiens*. The total targets of all the elements were combined after eliminating repeated targets. The follow-up analysis was conducted using the sum of the targets of all the elements.

### 4.4. Diabetes-Related Target Collection

The diabetes and glucose metabolism targets were gathered from the GeneCards database (retrieved from https://www.genecards.org/, accessed on 10 December 2024) [17]. For the following analysis, only the targets of protein-coding genes were chosen.

### 4.5. Overlapping Targets Between Components, Glucose Metabolism, and Diabetic Prediction

Using the Venny 2.1 online tool, the overlapping targets of diabetes, glucose metabolism, and components were plotted (https://bioinfogp.cnb.csic.es/tools/venny/, accessed on 15 December 2024) [18]. The overlapping area suggested potential targets in Chaga that have anti-diabetic effects via enhancing glucose metabolism.

### 4.6. Protein–Protein Interaction (PPI) Network Construction

STRING (Heidelberg, Germany) 12.0 was used to visualize the many different anti-diabetic targets of the components (https://string-db.org/, accessed on 15 December 2024). *Homo sapiens* was chosen for further analysis [19].

The PPI network was physically and functionally connected to the settings. The two nodes’ affiliations with one another were shown by the edge connecting them. Experiments, databases, co-expression, and other sources of interaction between nodes were all chosen. To enable identification, the overlapped nodes were separated. The PPI network was exported in two different formats: TSV and PNG.

### 4.7. Chaga Core Anti-Diabetic Targets for Enrichment Analysis Construction

The nodes whose degree centrality (DC), betweenness centrality (BC), and closeness centrality (CC) were all higher than the median were chosen as the primary targets after the interaction network was visualized using Cytoscape. Additionally, Metascape (https://metascape.org/gp/index.html#/main/step1, accessed on 20 December 2024) conducted the Kyoto Encyclopedia of Genes and Genomes (KEGG) and Gene Ontology (GO) enrichment analysis of the key targets [20].

### 4.8. Interaction Network Construction

Cytoscape (USA) 3.10.1 was used to import the PPI network with 431 targets that had been exported from STRING [54]. CytoNCA was utilized to compute the degree, betweenness, and closeness centralities for each of the 431 nodes. Additionally, cytoHubba was used to compute the DC, CC, maximum neighborhood component (MNC), and maximal clique centrality (MCC).

### 4.9. Chaga Central Targets for Component–Target Docking Construction

CytoHubba (Hsinchu, Taiwan, China) 0.1 was used to rank the 10 targets with the highest DC, CC, MCC, and MNC. The core targets for further docking were those that overlapped [55].

### 4.10. GO and KEGG Enrichment Analysis

The KEGG pathway enrichment analysis of cellular components (CCs), molecular functions (MFs), and biological processes (BPs) of core targets was all conducted on Metascape (retrieved from https://metascape.org/gp/index.html#/main/step1, accessed on 20 December 2024). *Homo sapiens* was selected as the organism. The *p*-value threshold was 0.01, the minimum enrichment was 1.5, and the minimum overlap was 3. Bioinformatics was used to visualize the findings of the enrichment study (https://www.bioinformatics.org/, accessed on 20 December 2024).

### 4.11. Chaga-Components-Targets-Diabetics-Signaling Pathway Network Construction

Cytoscape 3.10.1 was used to visualize the Chaga-components-targets-diabetics-signaling pathway interaction network. For convenience of presentation, the PubChem CID was used in place of the compound name. Different colors and forms were applied to nodes belonging to different groups. The components, targets, and pathways were arranged in a cyclic pattern based on their degree of centrality. Nodes with a higher degree of centrality were positioned closer to the center.

### 4.12. Molecular Docking

The study confirmed the interaction between central targets and five active phytochemicals from Chaga using molecular docking with the highest degree values [56]. Avoiding false-positive results from network pharmacology analysis is the goal of molecular docking. ChemDraw (version 23.0) was used to convert the active phytochemical structures into SMILES once they were downloaded from the PubChem database (https://pubchem.ncbi.nlm.nih.gov/, accessed on 3 January 2025). The central targets’ structure files were stored in PDB format after being retrieved from the PDB database (https://www.rcsb.org/, accessed on 3 January 2025). AutoDockTools was used to import the central targets and bioactive phytochemicals for pre-docking processing. The Autodock Vina was also accustomed to docking. PyMol and DiscoveryStudio tools were used to visualize and analyze some of the docking results [57].

### 4.13. Core Gene Expression Analysis

GSE7014 is a gene expression data set of 26 skeletal muscle samples (20 samples from diabetes patients and 6 healthy subjects) analyzed using GPL570 [HG-U133_Plus_2] Affymetrix human genome U133 Plus 2.0 array. GSE7014 was used as a training data set to explore the difference in core target expression between the diabetes group and the healthy group [58]. Then, we compared the expression of core targets in liver cancer and healthy individuals in the GEPIA database (http://gepia.cancer-pku.cn/, accessed on 3 January 2025) [59].

### 4.14. Analysis of Immune Cell Infiltration

The CIBERSORT algorithm (http://cibersort.stanford.edu/, accessed on 14 January 2025) is a proper method to identify the relationship between the core targets and immune cells. Using the CIBERSORT algorithm, we explored the mechanisms linking the core targets to 22 subtypes of immune cells in diabetes samples. These subtypes represent the cellular composition of the immune microenvironment in diabetes, and Spearman correlation analysis was carried out on immune cells and genes [60].

## 5. Conclusions

This study elucidates the potential anti-diabetic mechanisms of *Inonotus obliquus* (Chaga) by integrating network pharmacology, molecular docking, and bioinformatics analyses. The results identified key bioactive compounds such as forskolin, epicatechin, lupenone, laetiposide G, and smilagenone, which may play pivotal roles in regulating glucose metabolism and insulin sensitivity. Key targets such as JUN, AKT1, ESR1, CASP3, TNF, SRC, and IL6 were identified as central regulators in diabetes-related pathways, with molecular docking confirming strong binding interactions between Chaga-derived phytochemicals and these targets. Functional enrichment analysis highlighted the involvement of critical signaling pathways, including PI3K/Akt, which is closely associated with glucose metabolism and insulin sensitivity. Additionally, bioinformatics analysis revealed significant dysregulation of ESR1, IL6, and SRC in diabetic conditions, further supporting their relevance in disease progression. While these findings provide a theoretical framework for Chaga’s anti-diabetic effects, experimental validation through in vitro and in vivo studies is crucial to confirm its therapeutic potential. Future research needs to be conducted focusing on deciphering the precise molecular mechanisms, bioavailability, and clinical applicability of Chaga-derived compounds to establish them as promising candidates for diabetes management.

## Figures and Tables

**Table 1:** Compounds identified in Chaga mushroom by UHPLC-QE-MS.

No.	Compound Name	Compound Structure	Group	Target Number	PubChem CID	MZ Value	Adduct Ions	Type	Formula	Retention Time (s)	MS2 (M/Z)	Peak Value	ppm
1	Tamarixetin	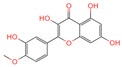	Flavone	109	5,281,699	315.0508719	[M-H]^−^	Neg	C1_6_H_12_O_7_	101.188	315.049869; 271.026506; 227.070704; 209.060198; 92.679913	423,008,185.4	0.406456701
2	Tectorigenin	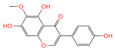	Isoflavone	73	5,281,811	299.0562603	[M-H]^−^	Neg	C_16_H_12_O_6_	155.009	299.055325; 255.066879; 211.0409; 237.054751; 227.035073	191,098,601.4	0.870455461
3	2-[6-(2-Carboxyethyl)-7-ethenyl-2-hydroxy-3a,6,9b-trimethyl-1,2,3,4,7,8-hexahydro cyclopenta[a]naphthalen-3-yl]-6-hydroxy-6-methyl-5-methylideneheptanoic acid	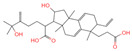	Polycyclic Aromatic Hydrocarbons, (PAHs)	54	163,043,082	499.3063976	[M-H]^−^	Neg	C_30_H_44_O_6_	467.32	499.30433; 187.097384; 92.680764; 125.096957; 500.31087	176,318,574	1.206423975
4	Alisol A	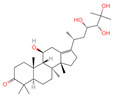	Triterpenoids	95	15,558,616	535.3637525	[M+FA]^−^	Neg	C_30_H_50_O_5_	489.307	535.37193; 489.361334; 536.364022; 386.850678; 109.065859	65,494,393.38	2.330227473
5	Asiatic acid	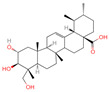	Triterpenoids	65	119,034	487.3445628	[M-H]^−^	Neg	C_30_H_48_O_5_	491.309	487.345071; 488.345858; 73.029652; 54.151807; 109.065719	114,915,667.3	1.154737219
6	Isosteviol	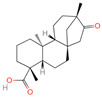	Diterpenoid	68	99,514	363.2182348	M+HCOO	Neg	C_20_H_30_O_3_	496.82	363.220266; 301.219918; 319.226219; 231.211763; 72.99321	26,364,667.26	0.646322331
7	Terminolic acid	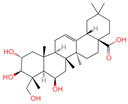	Triterpenoids glucoside	54	12,314,613	563.3608362	M+CH3COO	Neg	C_30_H_48_O_6_	520.801	563.351955; 485.331287; 517.353289; 582.091972; 103.881389	21,190,641.49	3.259305253
8	Arjungenin	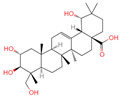	Triterpenoids	51	12,444,386	503.338039	[M-H]^−^	Neg	C_30_H_48_O_6_	526.846	503.338521; 92.68161; 425.302602; 457.29446; 485.260314	39,637,808.49	0.077564917
9	Methyl 4-(12-hydroxy-4,4,10,13,14-pentamethyl-3,7,11,15-tetraoxo-2,5,6,12,16,17-hexahydro-1H-cyclopenta[a]phenanthre-17-yl)pentanoate	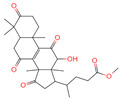	Triterpenoids	-	162,984,414	485.2549275	[M-H]^−^	Neg	C_28_H_38_O_7_	560.168	485.331281; 427.282211; 379.264977; 467.246817; 423.255523	34,371,858.3	0.149345426
10	Dehydrotumulosic acid	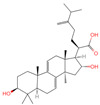	Triterpenoids	66	15,225,964	483.3487451	[M-H]^−^	Neg	C_31_H_48_O_4_	605.497	483.350109; 484.348208; 53.70788; 57.034681; 162.838479	13,338,969.54	1.54152815
11	Euscaphic acid	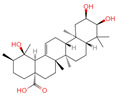	Triterpenoids	77	471,426	487.3420462	[M-H]^−^	Neg	C_30_H_48_O_5_	622.893	487.345165; 165.020038; 425.344197; 411.325271; 381.276225	439,249,992.8	1.957243167
12	Colosolic acid	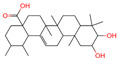	Triterpenoids	68	15,917,996	517.352961	[M+FA]^−^	Neg	C_30_H_48_O_4_	623.896	471.344563; 441.338464; 472.355581; 52.374559; 517.386766	19,924,369.48	2.008218219
13	Laetiposide G	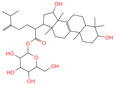	Triterpenoids	25	85,286,315	647.4166805	[M-H]^−^	Neg	C_37_H_60_O_9_	664.321	647.426051; 92.677381; 187.096644; 125.096959; 89.024806	6,672,290.148	0.493481417
14	2-[6-(2-Carboxyethyl)-7-ethenyl-3a,6,9b-trimethyl-1,2,3,4,7,8-hexahydro cyclopenta[a]naphthalen-3-yl]-6-methyl-5-methylideneheptanoic acid	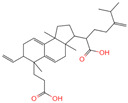	Triterpenoids	52	162,953,199	467.3161948	[M-H]^−^	Neg	C_30_H_44_O_4_	693.617	467.314009; 371.261375; 83.050558; 92.681611; 468.321422	20,388,189.76	1.722931034
15	Betulinic acid	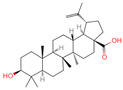	Triterpenoids	53	64,971	455.3526274	[M-H]^−^	Neg	C_30_H_48_O_3_	703.636	455.353083; 456.359141; 50.597266; 92.676531; 411.293613	581,078,510.3	3.01445483
16	Oleanonic acid	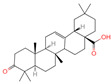	Triterpenoids	85	12,313,704	499.3432828	M+HCOO	Neg	C_30_H_46_O_3_	760.737	453.341453; 61.988561; 92.680765; 454.341248; 50.373094	9,760,177.404	0.566249293
17	Epicatechin	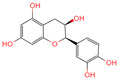	Flavanol	20	72,276	291.0861046	[M+H]^+^	Pos	C_15_H_14_O_6_	46.5308	139.038665; 123.044504; 165.054501; 147.043675; 291.087129	373,945,509.4	0.359428699
18	Glabrol	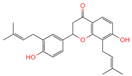	Flavanones	20	480,768	393.2089956	[M+H]^+^	Pos	C_25_H_28_O_4_	58.8721	393.205584; 394.213319; 92.657626; 153.054649; 375.102135	324,258,064.6	0.011256345
19	Forskolin	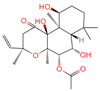	Diterpenoid	3	47,936	411.2381929	[M+H]^+^	Pos	C_22_H_34_O_7_	244.05	393.227554; 375.21174; 167.106581; 125.095873; 411.24093	775,007,987.6	0.469105286
20	Mimusopsic acid	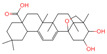	Triterpenoids	20	162,981,968	485.3253743	[M+H]^+^	Pos	C_30_H_44_O_5_	463.121	485.323255; 467.314296; 449.303379; 421.309481; 95.085504	1,676,895,247	1.289307824
21	Neokadsuranic acid B	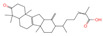	Triterpenoids	92	78,385,354	453.3374578	[M+H]^+^	Pos	C_30_H_44_O_3_	481.408	453.340042; 435.329506; 417.317354; 107.085173; 311.236845	302,123,599.7	3.215767303
22	Smilagenone	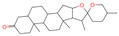	Triterpenoids	22	313,275	415.320709	[M+H]^+^	Pos	C_27_H_42_O_3_	485.857	415.063669; 397.313404; 109.100875; 95.085575; 119.085774	25,172,111.45	0.700548339
23	18 beta-Glycyrrhetintic Acid	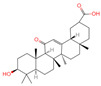	Triterpenoids	81	10,114	471.3468656	[M+H]^+^	Pos	C_30_H_46_O_4_	513.041	471.344968; 453.343313; 107.085123; 95.085453; 435.329815	917,819,007.6	0.285164639
24	3beta-hydroxy-21-oxo-11,13(18)-oleanadien-28-oic acid methyl ester	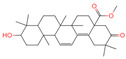	Triterpenoids	43	163,035,166	483.3465734	[M+H]^+^	Pos	C_31_H_46_O_4_	521.46	483.311442; 465.288031; 429.277318; 405.318836; 447.283351	69,113,720.32	0.882538726
25	Wilforlide A	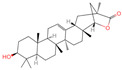	Triterpenoids	48	158,477	455.3517334	[M+H]^+^	Pos	C_30_H_46_O_3_	528.872	455.357056; 81.069528; 437.352349; 109.100779; 69.069803	513,163,312.1	0.585490831
26	Ganoderic aldehyde A	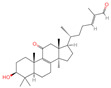	Triterpenoids	84	163,036,286	453.3356962	[M+H]^+^	Pos	C_30_H_44_O_3_	642.652	453.331046; 435.322148; 69.069814; 109.100811; 81.069578	167,072,813.4	2.876074492
27	Dehydrotrametenolic acid	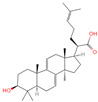	Triterpenoids	56	15,391,340	455.3514154	[M+H]^+^	Pos	C_30_H_46_O_3_	647.617	455.351776; 107.08527; 109.100787; 121.101364; 95.08478	250,319,010.4	1.283864243
28	Betulin	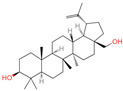	Triterpenoids	31	72,326	443.3792757	[M+H]^+^	Pos	C_30_H_50_O_2_	672.397	443.311471; 69.0698; 425.306414; 109.100714; 81.069471	71,825,700.8	21.93225228
29	Lupenone	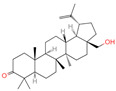	Triterpenoids	22	92,158	425.3770963	[M+H]^+^	Pos	C_30_H_48_O	714.536	95.084839; 81.069561; 69.069828; 137.132042; 425.372928	1,002,461,661	2.124550292
30	Panaxatriol	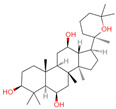	Triterpene sapogenin	89	73,599	459.3823159	M+H-H_2_O	Pos	C_30_H_52_O_4_	743.23	459.377958; 441.371333; 423.359272; 135.116679; 69.069876	42,410,492.67	1.489083716

## Data Availability

The datasets used and analyzed in the current study are available from the corresponding author on reasonable request.

## Abbreviations

AGEs	Advanced glycation end-products
Akt	Protein kinase B
AMPK	AMP-activated protein kinase
BC	Betweenness centrality
BPs	Biological processes
CC	Closeness centrality
CCs	Cellular components
DC	Degree of centrality
DM	Diabetes mellitus
GLUT4	Glucose transporter type 4
GO	Gene Ontology
GSK-3β	Glycogen synthase kinase-3β
KEGG	Kyoto Encyclopedia of Genes and Genomes
LC-MS	Liquid chromatography–mass spectrometry
MCC	Maximal clique centrality
MFs	Molecular functions
MNC	Maximum neighborhood component
PI3K/Akt	Phosphatidylinositol 3-kinase/protein kinase B
PPI	Protein–protein interaction
ROS	Reactive oxygen species
T2D	Type 2 diabetes
UHPLC	Ultra-high-performance liquid chromatography
UHPLC-QE-MS	Ultra-high-performance liquid chromatography–Q-Exactive HF mass spectrometry

## References

[1] International Diabetes Federation (2021). IDF Diabetes Atlas.

[2] Li M., Chi X., Wang Y., Setrerrahmane S., Xie W., Xu H. (2022). Trends in insulin resistance: Insights into mechanisms and therapeutic strategy. Signal Transduct. Target. Ther..

[3] Huang X., Liu G., Guo J., Su Z. (2018). The PI3K/AKT pathway in obesity and type 2 diabetes. Int. J. Biol. Sci..

[4] González P., Lozano P., Ros G., Solano F. (2023). Hyperglycemia and oxidative stress: An integral, updated and critical overview of their metabolic interconnections. Int. J. Mol. Sci..

[5] Abugomaa A., Elbadawy M., Ishihara Y., Yamamoto H., Kaneda M., Yamawaki H., Sasaki K. (2023). Anti-cancer activity of Chaga mushroom (*Inonotus obliquus*) against dog bladder cancer organoids. Front Pharmacol..

[6] Doi N., Araki K., Fukuta Y., Kuwagaito Y., Yamauchi Y., Sasai Y., Kuzuya M. (2023). Anti-glycation and antioxidant effects of Chaga mushroom decoction extracted with a fermentation medium. Food Sci. Tech. Res..

[7] Alhallaf W., Perkins L.B. (2022). The anti-inflammatory properties of chaga extracts obtained by different extraction methods against LPS-induced RAW 264.7. Molecules.

[8] Er Demirhan B., Demirhan B. (2022). Investigation of twelve significant mycotoxin contamination in nut-based products by the LC–MS/MS method. Metabolites.

[9] Xu X., Pang C., Yang C., Zheng Y., Xu H.Y., Lu Z., Xu Z.H. (2010). Antihyperglycemic and antilipidperoxidative effects of polysaccharides extracted from medicinal mushroom Chaga, *Inonotus obliquus* (Pers.: Fr.) Pilat (Aphyllophoromycetideae) on alloxan-diabetes mice. Int. J. Med. Mushrooms..

[10] Shen D., Feng Y., Zhang X., Liu J., Gong L., Liao H., Li R. (2022). In Vitro Immunomodulatory Effects of *Inonotus obliquus* Extracts on Resting M0 Macrophages and LPS-Induced M1 Macrophages. Evid. Based Complement. Alternat Med..

[11] Szychowski K.A., Skóra B., Pomianek T., Gmiński J. (2020). *Inonotus obliquus*—From folk medicine to clinical use. J. Trad. Complement. Med..

[12] Aramabašić Jovanović J., Mihailović M., Uskoković A., Grdović N., Dinić S., Vidaković M. (2021). The effects of major mushroom bioactive compounds on mechanisms that control blood glucose level. J. Fungi.

[13] Feng Y., Ren Y., Zhang X., Yang S., Jiao Q., Li Q., Jiang W. (2024). Metabolites of traditional Chinese medicine targeting PI3K/AKT signaling pathway for hypoglycemic effect in type 2 diabetes. Front. Pharmacol..

[14] Wang J., Wang C., Li S., Li W., Yuan G., Pan Y., Chen H. (2017). Anti-diabetic effects of *Inonotus obliquus* polysaccharides in streptozotocin-induced type 2 diabetic mice and potential mechanism via PI3K-Akt signal pathway. Biomed. Pharmacother..

[15] Daina A., Michielin O., Zoete V. (2019). SwissTargetPrediction: Updated data and new features for efficient prediction of protein targets of small molecules. Nucleic Acids Res..

[16] Keiser M.J., Roth B.L., Armbruster B.N., Ernsberger P., Irwin J.J., Shoichet B.K. (2007). Relating protein pharmacology by ligand chemistry. Nat. Biotech..

[17] Safran M., Rosen N., Twik M., BarShir R., Stein T.I., Dahary D., Lancet D. (2021). The genecards suite. Practical Guide to Life Science Databases.

[18] Oliveros J.C. (2007). VENNY. An Interactive Tool for Comparing Lists with Venn Diagrams. http://bioinfogp.cnb.csic.es/tools/venny/index.html.

[19] Szklarczyk D., Kirsch R., Koutrouli M., Nastou K., Mehryary F., Hachilif R., Gable A.L., von Mering C. (2023). The STRING database in 2023: Protein-protein association networks and functional enrichment analyses for any sequenced genome of interest. Nucleic Acids Res..

[20] Zhou Y., Zhou B., Pache L., Chang M., Khodabakhshi A.H., Tanaseichuk O., Benner C., Chanda S.K. (2019). Metascape provides a biologist-oriented resource for the analysis of systems-level datasets. Nat. Commun..

[21] Liu Y., Zhang J., Liu X., Zhou W., Stalin A., Fu C., Lu S. (2021). Investigation on the mechanisms of guiqi huoxue capsule for treating cervical spondylosis based on network pharmacology and molecular docking. Medicine.

[22] Wong F., Krishnan A., Zheng E.J., Stärk H., Manson A.L., Earl A.M., Jaakkola T., Collins J.J. (2022). Benchmarking AlphaFold-enabled molecular docking predictions for antibiotic discovery. Mol. Syst. Biol..

[23] Luo J., Luo J., Wu Y., Fu Y., Fang Z., Han B., Du B., Yang Z., Xu B. (2024). Anti-obesity effects of Adzuki bean saponins in improving lipid metabolism through reducing oxidative stress and alleviating mitochondrial abnormality by activating the PI3K/Akt/GSK3β/β-catenin signaling pathway. Antioxidants.

[24] Liu Y., Luo J., Xu B. (2024). Elucidation of anti-obesity mechanisms of phenolics in *Artemisiae argyi folium* (aiye) by integrating LC-MS, network pharmacology, and molecular docking. Life.

[25] Han B., Luo J., Xu B. (2024). Revealing molecular mechanisms of the bioactive saponins from edible root of Platycodon grandiflorum in combating obesity. Plants.

[26] Liu Y., Luo J., Meenu M., Xu B. (2025). Anti-obesity mechanisms elucidation of essential oil components from *Artemisiae Argyi Folium* (Aiye) by the integration of GC-MS, network pharmacology, and molecular docking. Int. J. Food Prop..

[27] Luo J., Wu Y., Moussa A.Y., Huang Y., Fu Y., Wei Y., Xu B. (2024). Unveiling the molecular mechanisms of adzuki bean (*Vigna angularis*) derived bioactive phytochemicals in combating obesity. Food Biosci..

[28] Ern P.T.Y., Quan T.Y., Yee F.S., Yin A.C.Y. (2023). Therapeutic properties of *Inonotus obliquus* (Chaga mushroom): A review. Mycology.

[29] Luo J., Yan W., Chen Z., Xu B. (2024). Elucidating the anti-obesity phytochemicals in Chenpi and their molecular mechanisms. Food Sci. Human Wellness.

[30] Kiselova-Kaneva Y., Galunska B., Nikolova M., Dincheva I., Badjakov I. (2022). High resolution LC-MS/MS characterization of polyphenolic composition and evaluation of antioxidant activity of Sambucus ebulus fruit tea traditionally used in Bulgaria as a functional food. Food Chem..

[31] Saini R.K., Song M.H., Yu J.W., Lee J.H., Ahn H.Y., Keum Y.S., Lee J.H. (2022). Profiling of nutritionally vital bioactive compounds in emerging green leafy vegetables: A comparative study. Foods.

[32] Agu P.C., Afiukwa C.A., Orji O.U., Ezeh E.M., Ofoke I.H., Ogbu C.O., Ugwuja E.I., Aja P.M. (2023). Molecular docking as a tool for the discovery of molecular targets of nutraceuticals in diseases management. Sci. Rep..

[33] Wang K., Yin J., Chen J., Ma J., Si H., Xia D. (2024). Inhibition of inflammation by berberine: Molecular mechanism and network pharmacology analysis. Phytomedicine.

[34] Nauck M.A., Müller T.D. (2023). Incretin hormones and type 2 diabetes. Diabetologia.

[35] Mironov N., Haque M., Atfi A., Razzaque M.S. (2022). Phosphate dysregulation and metabolic syndrome. Nutrients.

[36] Erekat N.S. (2022). Programmed cell death in diabetic nephropathy: A review of apoptosis, autophagy, and necroptosis. Int. Med. J. Experimen Clin. Res..

[37] Saltiel A.R. (2021). Insulin signaling in health and disease. J. Clin. Investig..

[38] Zhao X., An X., Yang C., Sun W., Ji H., Lian F. (2023). The crucial role and mechanism of insulin resistance in metabolic disease. Front. Endocrinol..

[39] Das D., Sarkar S., Dihingia A., Afzal N.U., Wann S.B., Kalita J., Dewanjee S., Manna P. (2022). A popular fermented soybean food of Northeast India exerted promising antihyperglycemic potential via stimulating PI3K/AKT/AMPK/GLUT4 signaling pathways and regulating muscle glucose metabolism in type 2 diabetes. J. Food Biochem..

[40] Zhou Y.J., Xu N., Zhang X.C., Zhu Y.Y., Liu S.W., Chang Y.N. (2021). Chrysin improves glucose and lipid metabolism disorders by regulating the AMPK/PI3K/AKT signaling pathway in insulin-resistant HepG2 cells and HFD/STZ-induced C57BL/6J mice. J. Agric. Food Chem..

[41] Fazakerley D.J., Koumanov F., Holman G.D. (2021). GLUT4 On the move. Biochem. J..

[42] Jin D.X., He J.F. (2022). Pi3k/akt signaling pathway–mediated three flavonoids’ modulation on glucose metabolism. Rev. Bras. Farmacogn..

[43] Liu Y., Qiu Y., Chen Q., Han X., Cai M., Hao L. (2021). Puerarin suppresses the hepatic gluconeogenesis via activation of PI3K/Akt signaling pathway in diabetic rats and HepG^2^ cells. Biomed. Pharmacother..

[44] Chen J.Y., Peng S.Y., Cheng Y.H., Lee I.T., Yu Y.H. (2021). Effect of Forskolin on body weight, glucose metabolism and adipocyte size of diet-induced obesity in mice. Animals.

[45] Naghibi M., Tayefi Nasrabadi H., Soleimani Rad J., Garjani A., Gholami Farashah M.S., Mohammadnejad D. (2023). Forskolin improves male reproductive complications caused by hyperglycemia in type 2 diabetic rats. Int. J. Fertil. Steril..

[46] Fraga C.G., Cremonini E., Galleano M., Oteiza P.I. (2025). Natural products and diabetes: (-)-epicatechin and mechanisms involved in the regulation of insulin sensitivity. Handb. Exp. Pharmacol..

[47] Slavova-Kazakova A., Janiak M.A., Sulewska K., Kancheva V.D., Karamać M. (2021). Synergistic, additive, and antagonistic antioxidant effects in the mixtures of curcumin with (-)-epicatechin and with a green tea fraction containing (-)-epicatechin. Food Chem..

[48] Lakthan T., Limpachayaporn P., Rayanil K.O., Charoenpanich P., Phuangbubpha P., Charoenpanich A. (2023). Lupenone-rich fraction derived from *Cissus quadrangularis* L. suppresses lipid accumulation in 3T3-L1 adipocytes. Life.

[49] Yung J.H.M., Giacca A. (2020). Role of c-Jun N-terminal kinase (JNK) in obesity and type 2 diabetes. Cells.

[50] Taheri R., Mokhtari Y., Yousefi A.M., Bashash D. (2024). The PI3K/Akt signaling axis and type 2 diabetes mellitus (T2DM): From mechanistic insights into possible therapeutic targets. Cell Biol. Int..

[51] Gregorio K.C.R., Laurindo C.P., Machado U.F. (2021). Estrogen and glycemic homeostasis: The fundamental role of nuclear estrogen receptors ESR1/ESR2 in glucose transporter GLUT4 regulation. Cells.

[52] O’Brien M.H., Pitot H.C., Chung S.H., Lambert P.F., Drinkwater N.R., Bilger A. (2021). estrogen receptor-α suppresses liver carcinogenesis and establishes sex-specific gene expression. Cancers.

[53] Zhang Y.Y., Elam E., Ni Z.J., Zhang F., Thakur K., Wang S., Zhang J.G., Wei Z.J. (2022). LC-MS/MS targeting analysis of terpenoid metabolism in *Carya cathayensis* at different developmental stages. Food Chem..

[54] Shannon P., Markiel A., Ozier O., Baliga N.S., Wang J.T., Ramage D., Amin N., Schwikowski B., Ideker T. (2003). Cytoscape: A software environment for integrated models of biomolecular interaction networks. Genome Res..

[55] Liu L., Jiao Y., Yang M., Wu L., Long G., Hu W. (2023). Network pharmacology, molecular docking and molecular dynamics to explore the potential immunomodulatory mechanisms of deer antler. Int. J. Mol. Sci..

[56] Zhao S., Ni F., Qiu T., Wolff J.T., Tsai S.C., Luo R. (2020). Molecular Basis for Polyketide Ketoreductase-Substrate Interactions. Int. J. Mol. Sci..

[57] PyMOL (2017). The PyMOL Molecular Graphics System, Version 2.0.

[58] Wang W., Wang Y. (2023). Integrative bioinformatics analysis of biomarkers and pathways for exploring the mechanisms and molecular targets associated with pyroptosis in type 2 diabetes mellitus. Front. Endocrinol..

[59] Tang Z., Li C., Kang B., Gao G., Li C., Zhang Z. (2017). GEPIA: A web server for cancer and normal gene expression profiling and interactive analyses. Nucleic Acids Res..

[60] Zhou Y., Liu C., Zhang Z., Chen J., Zhao D., Li L., Tong M., Zhang G. (2023). Identification and validation of diagnostic biomarkers of coronary artery disease progression in type 1 diabetes via integrated computational and bioinformatics strategies. Comput. Biol. Med..

